# Speculating suitability of partial adrenalectomy for lateralized primary aldosteronism: With emphasis on partial and complete success as optimistic outcomes

**DOI:** 10.1038/s41440-025-02101-6

**Published:** 2025-03-03

**Authors:** Tsae-Ni Lee, Chin-Chen Chang, Jeff S. Chueh, Chi-Shin Tseng, Vin-Cent Wu, Kang-Yung Peng, Po-Lung Yang, Shuo-Meng Wang, TAIPAI (Taiwan Primary Aldosteronism Investigation) study group, Vin-Cent Wu, Tai-Shuan Lai, Shih-Chieh Jeff Chueh, Shao-Yu Yang, Kao-Lang Liu, Chin-Chen Chang, Bo-Chiag Lee, Shuo-Meng Wang, Kuo-How Huang, Po-Chih Lin, Yen-Hung Lin, Chi-Sheng Hung, Lian-Yu Lin, Shih-Cheng Liao, Ching-Chu Lu, Chieh-Kai Chan, Leay-Kiaw Er, Ya-Hui Hu, Che-Hsiung Wu, Yao-Chou Tsai, Zheng-Wei Chen, Chien-Ting Pan, Che-Wei Liao, Cheng-Hsuan Tsai, Yi-Yao Chang, Chen-Hsun Ho, Wei-Chieh Huang, Ying-Ying Chen

**Affiliations:** 1 Department of Urology, National Taiwan University Hospital, Taipei, Taiwan; 2 Department of Radiology, National Taiwan University Hospital, Taipei, Taiwan; 3 Division of Nephrology, Department of Internal Medicine, National Taiwan University Hospital, Taipei, Taiwan; 4 National Taiwan University Hospital, Taipei, Taiwan; 5 Department of Internal Medicine, National Taiwan University Hospital Hsinchu Branch, Hsinchu, Taiwan; 6 Taipei Tzu-Chi Hospital, Buddhist Tzu Chi Medical Foundation, Taipei, Taiwan; 7 Department of Internal Medicine, National Taiwan University Hospital Yunlin Branch, Yunlin, Taiwan; 8 Department of Medicine, National Taiwan University Cancer Center, Taipei, Taiwan; 9 Department of Cardiovascular Medicine, Far Eastern Memorial Hospital, New Taipei City, Taiwan; 10 Department of Urology, Shin Kong Wu Ho-Su Memorial Hospital, Taipei, Taiwan; 11 Department of Internal Medicine, Taipei Veterans General Hospital, Taipei, Taiwan; 12 Department of Internal Medicine, MacKay Memorial Hospital, Taipei, Taiwan

**Keywords:** Primary aldosteronism, Hypertension, Adrenalectomy, Adenoma, CYP11B2

## Abstract

Primary aldosteronism (PA) is the most common secondary hypertension. The best treatment for a lateralized PA is unilateral adrenalectomy. Recent studies explored partial adrenalectomy (pAdx) to reduce the risk of adrenal insufficiency. However, in cases involving multiple aldosterone-producing micronodules/nodules (mAPM/mAPN), pAdx cannot completely remove all origins of excess aldosterone and might not resolve hypertension. CYP11B2 immunohistochemical staining helps HISTALDO (Histopathology of PA) diagnosis, and adrenal specimens were categorized into various groups accordingly. To determine whether pAdx should be considered for lateralized PA, we focused on the success rate of classical (black + grey group) versus non-classical (white group) lateralized PA, and the percentage of co-existing mAPM/mAPN in lateralized PA. The visible tumor in imaging could be either non-functional (incidentaloma; white group), or with concurrent surrounding mAPM/mAPN (grey group) causing hypertension. Among 445 patients who underwent unilateral adrenalectomy, 390 were diagnosed with lateralized PA. There were 63 (30.73%) in the black, 79 (38.54%) in the grey, 63 (30.73%) in the white group. The overall complete clinical success rate was 51.28% in our lateralized PA patients; with 65.08% in the black, 50.63% in the grey, and 26.98% in the white group. The overall partial clinical success rate was 38.54%; with 28.57% in the black, 34.18% in the grey, and 53.97% in the white group. Were pAdx performed, significantly lower success rates would be achieved, especially for lateralized PA patients of the grey and white groups. We speculate that unilateral pAdx is not an appropriate option for the majority of lateralized PA patients.

**Figure:**
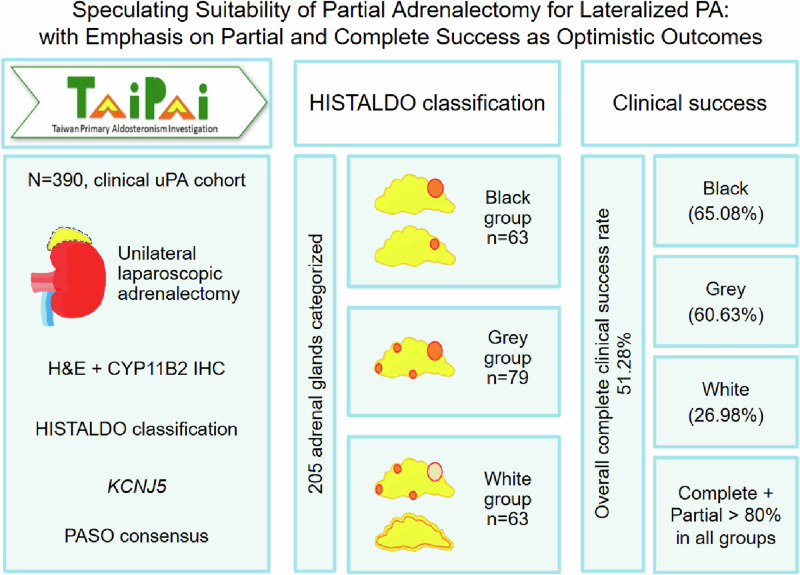
Our results show that unilateral partial adrenalectomy is not a good surgical option for the majority of lateralized PA patients. In clinically lateralized PA patients, no matter which group they are in the HISTALDO classification, they would benefit from unilateral total adrenalectomy.

Our results show that unilateral partial adrenalectomy is not a good surgical option for the majority of lateralized PA patients. In clinically lateralized PA patients, no matter which group they are in the HISTALDO classification, they would benefit from unilateral total adrenalectomy.

## Abstract (web summary)

Our results show that unilateral partial adrenalectomy is not a good surgical option for the majority of lateralized PA patients. In clinically lateralized PA patients, no matter which group they are in the HISTALDO classification, they would benefit from unilateral total adrenalectomy.

## Introduction

Primary aldosteronism (PA), the most common cause of secondary hypertension, is characterized by excessive endogenous plasma aldosterone production in the presence of suppressed plasma renin activity (PRA) [1, 2]. The treatment of choice for a lateralized PA is unilateral adrenalectomy (surgical removal of the affected adrenal gland), which aims to resolve excess aldosterone production and, subsequently, alleviate hypertension; potentially allowing PA patients to discontinue (all) antihypertensive medications [3].

Traditionally, total adrenalectomy (complete removal of the affected unilateral adrenal gland) was the standard approach. However, recent studies have explored the feasibility and potential benefits of a partial adrenalectomy, which involves removing only the part of the adrenal gland that contains the tumoral mass, while preserving the rest of the grossly normal-looking gland. By sparing a portion of the adrenal gland, PA patients might be at a lower risk of developing future adrenal insufficiency, which could be a concern after total adrenalectomy [4]. Partial adrenalectomy is often considered in cases where the adrenal tumor is small, likely benign, and not cancerous [5], or in cases of simultaneous or metachronous bilateral adrenal tumors. However, in some PA cases, especially those involving multiple aldosterone-producing micronodules/nodules (mAPM/mAPN), partial adrenalectomy cannot completely remove the origins of all excessive aldosterone-producing tissues, and thus might be unable to resolve hypertension, and defeat the initial purpose of the surgery [6]. There is a possibility that the main tumoral mass shown in imaging studies is either non-functional (an incidentaloma) or only partially functional, with concurrent mAPM/mAPN in the surrounding healthy-looking adrenal gland still leading to residual hypertension [7–10].

Cytochrome P450 11B2 (CYP11B2), also known as aldosterone synthase, is essential in aldosterone synthesis and plays an important role in the pathogenesis of PA [11]. By using immunochemistry with specific monoclonal antibodies targeting CYP11B2, cells that synthesize aldosterone in the adrenal zona glomerulosa can be identified [12]. The expression of CYP11B2 is typically regulated by angiotensin II, however, autonomous CYP11B2 expression is seen in aldosterone-producing adenomas (APA), aldosterone-producing nodules (APN) or mPAM/mAPN, or even aldosterone-producing diffuse hyperplasia (APDH), which could possibly be due to somatic mutations of certain potassium or calcium channel, with mutations of the gene encoding the potassium inwardly rectifying channel, subfamily J, member 5 (*KCNJ5*) being the most common [13–17]. Whereas CYP11B1, the gene encoding 11-beta-hydroxylase, is important in cortisol synthesis. Studies revealed lower CYP11B1 and higher CYP11B2 expression in adrenal adenomas in PA than those in Cushing’s syndrome [18]. CYP11B2 immunohistochemical staining is a robust method for the histopathological diagnosis and classification of primary aldosteronism (HISTALDO; histopathology of PA) [19].

This study aims to determine the proportion of classical (black group + grey group) and non-classical (white group) lateralized PA, the percentage of co-existing mAPM/mAPN in clinically diagnosed lateralized PA, and the clinical and biochemical outcomes after undergoing a total adrenalectomy in each groups, according to the PA surgical outcomes criteria (PASO) [20, 21].

Point of view

- **Clinical relevance**
  Partial adrenalectomy is not a good surgical option for the majority of lateralized PA patient. Clinically lateralized PA patients would benefit from unilateral total adrenalectomy, no matter which group they are in the HISTALDO classification. Having a positive *KCNJ5* mutation does not assure surgical success.
- **Future direction**
  Further, fully prospective evaluation to elucidate the clinical and pathophysiological significance of adrenalectomy and *KCNJ5* mutation on lateralized PA patients is warranted.
- **Consideration for the Asian population**
  While the presence of a *KCNJ5* somatic mutation has been identified as an independent predictor of hypertension remission following unilateral adrenalectomy in patients with lateralized PA, its prevalence differs between Asian and European populations.

## Materials & methods

This study was conducted by investigators from the TAIPAI (Taiwan Primary Aldosteronism Investigation) study group via using a prospective collection of patients and specimens with PA.

From February 1999 to August 2018, patients with hypertension and an image-identifiable adrenal nodule/adenoma who underwent unilateral laparoscopic adrenalectomy were enrolled. The diagnosis of PA was made via standardized aldosterone/renin ratio screening test and confirmatory tests, including the saline infusion test or captopril test [22]. Adrenal venous sampling (AVS) was performed to assure its functional unilateral lateralization. APA is further confirmed after adrenalectomy: pathologically proven a CYP11B2 adenoma or aldosterone-producing cell clusters at immunohistochemistry after adrenalectomy, and subsequent emergence of biochemical correction. Standard H&E staining and immunohistochemistry staining using a mouse monoclonal antibody for CYP11B2 were analyzed retrospectively.

The lateralization index (LI) of AVS is defined as the ratio of the aldosterone/cortisol concentration on the dominant side to that on the contralateral side. Successful AVS is defined as a selectivity index (SI) value ≥ 2.0 bilaterally; in which SI is defined as the ratio of the sampled cortisol concentration of each adrenal vein to that of the peripheral vein without the stimulation of cosyntropin. After confirming successful bilateral AVS, confirmative lateralization of the PA was determined by an LI value ≥ 2.0.

The excised adrenal specimens were categorized into three groups according to the HISTALDO criteria, including: “black group” for solitary APA or dominant APN with strong positive CYP11B2 staining in the tumor mass only, “grey group” for cases with both the main tumor and mAPM/mAPN in the surrounding H&E normal-looking adrenal tissues showing positive CYP11B2 staining; and “white group” for the main tumor with negative CYP11B2 staining (incidentaloma) and surrounding mAPM/mAPN with positive CYP11B2 staining. The PA patients in the black and grey groups form the “classical” lateralized PA patients, and the PA patients in the white groups consist of the “non-classical” lateralized PA patients [8].

Clinical and biochemical outcomes were defined based on the PASO consensus, with at least 2 post-adrenalectomy follow-up visits. “Complete clinical success” was defined according to the PASO criteria of normalized blood pressure without any antihypertensive agents after adrenalectomy. “Complete biochemical success” was defined as normalized aldosterone-renin ratio (ARR) and normalized potassium level after adrenalectomy. “Partial clinical success” was defined according to the PASO criteria of better blood pressure control with still some antihypertensive agents needed after adrenalectomy [20]. The existence of a *KCNJ5* somatic mutation was checked with Sanger or next-generation sequencing technique from the excised adenomas/nodules or CYP11B2-positive adrenal tissues.

### Statistical analysis

All statistical analyses were tabulated and analyzed by Stata version 16 for Windows® (Stata Corp, College Station, TX, USA). The Pearson’s chi‑square test and Fisher’s exact test were used to evaluate the association between categorical variables, and the ANOVA test was applied to compare the continuous variables. The changes of the preoperative and postoperative blood pressure were analyzed using paired *t*-test. All tests were two-sided with *p* < 0.05 considered statistically significant. Post-hoc test was performed with Bonferonni correction.

## Results

**Fig. 1:**
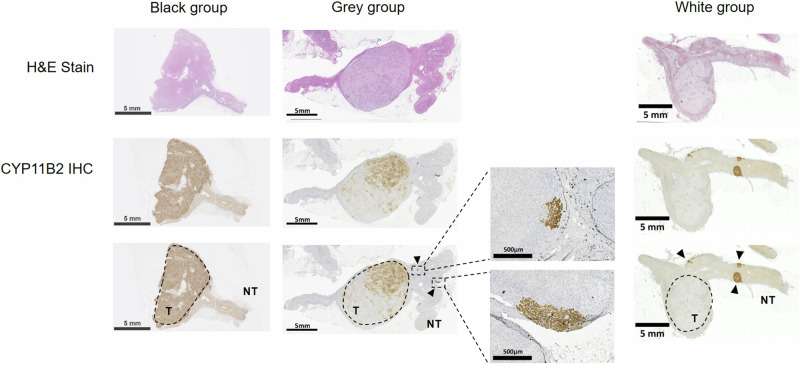
Histopathological and immunohistochemistry (CYP11B2) features of the black (solitary APA or dominant APN with strong positive CYP11B2 staining in the tumor mass only), grey (both the main tumoral mass and mAPM/mAPN in the surrounding H&E normal-looking adrenal tissues showing positive CYP11B2 staining), and white (main tumor with negative CYP11B2 staining (incidentaloma) and surrounding mAPM/mAPN with positive CYP11B2 staining) groups

A total of 445 patients who underwent unilateral laparoscopic adrenalectomy were enrolled. Amongst them, 390 patients were diagnosed with lateralized PA. Among the 390 lateralized PA patients, 174 (44.6%) were men and 216 (55.4%) were women. The mean age was 48.4, 50.6, and 54.2 years old in the black, grey, and white groups, respectively. There was no significance regarding gender, mean body weight, mean body height, mean body mass index (BMI), mean waist line, mean buttocks width, mean blood pressure, duration of hypertension, Charlson comorbidity index, maximum diameter of the adrenal nodule, AVS LI, nor contralateral suppression during adrenal venous sampling among the three groups. The number of antihypertensive agents used preoperatively and the preoperative ARR were comparable between groups (Table 1). The histopathological features and immunohistochemistry findings of the black group (solitary APA or a dominant APN with positive CYP11B2 staining), grey group (both the main tumor and mAPM/mAPN showing positive CYP11B2 staining), and white group (main tumor with negative CYP11B2 staining and mAPM/mAPN with positive CYP11B2 staining) are shown in Fig. 1. The resected adrenal glands were categorized into three groups according to the HISTALDO criteria: 63 (30.73%) in the black group, 79 (38.54%) in the grey group, 63 (30.73%) in the white group; whereas 185 adrenal glands/tumors were too challenging to be classified due to limited specimens.

**Table 1:** Demographics of the PA patients in the black, grey, and white groups

	Black (*n* = 63)	Grey (*n* = 79)	White (*n* = 63)	*p*-value
Mean age, years (SD)	48.4 (10.9)	50.6 (10.6)	54.2 (11.6)	0.0125
Males, *n* (%)	35 (55.6%)	36 (45.6%)	29 (46.0%)	0.433
Mean body weight, kg (SD)	69.0 (14.1)	69.2 (16.8)	69.0 (15.0)	0.9948
Mean body height, cm (SD)	165.7 (8.8)	164.1 (9.0)	163.5 (9.0)	0.3477
Mean BMI, kg/m2 (SD)	25.0 (4.0)	25.5 (4.5)	25.5 (3.9)	0.7223
Mean waist line, cm (SD)	84.9 (11.4)	85.6 (13.5)	84.9 (11.9)	0.931
Mean buttocks width, cm (SD)	96.8 (7.3)	97.1 (11.1)	96.8 (9.4)	0.9674
Mean blood pressure, mmHg (SD)				
Systolic	157.9 (22.7)	154.9 (19.4)	150.2 (19.0)	0.1059
Diastolic	95.5 (14.2)	92.3 (12.3)	90.2 (13.5)	0.0869
Medical history, *n* (%)				
DM	4 (6.35%)	14 (17.72%)	10 (15.87%)	0.103
Hyperlipidemia	11 (17.46%)	19 (24.05%)	18 (28.57%)	0.333
Hyperthyroidism	0	1 (1.27%)	0	1
Hypothyroidism	1 (1.59%)	0	2 (3.17%)	0.286
Atrial fibrillation	2 (3.17%)	3 (3.80%)	1 (1.59%)	0.877
CAD/MI	0	0	0	
CVA	4 (6.35%)	5 (6.33%)	4 (6.35%)	1
Renal stone	6 (9.52%)	5 (6.33%)	3 (4.76%)	0.612
Smoking history	7 (11.11%)	5 (6.33%)	11 (17.46%)	0.114
Family history of HTN	35 (55.56%)	60 (75.95%)	44 (69.84%)	0.033
Duration of HTN, year(s) (SD)	5.8 (6.6)	8.2 (7.1)	8.4 (8.7)	0.084
CCI (SD)	1.5 (1.5)	1.5 (1.3)	1.5 (1.4)	0.9692
AVS LI (SD)	4.61 (6.35)	5.44 (6.43)	2.91 (3.43)	0.3721
Contralateral suppression, *n* (%)	3 (4.8%)	2 (2.53%)	0	0.277
Maximum adrenal nodule diameter, cm (SD)	1.9 (1.2)	1.8 (1.1)	1.7 (0.7)	0.84
Mean number of antihypertensive agents, *n* (SD)	2.0 (1.3)	2.2 (1.1)	1.9 (1.3)	0.3229
ACEI, *n* (%)	2 (3.2%)	0	0	0.187
α-blocker, *n* (%)	18 (28.6%)	16 (20.3%)	14 (22.2%)	0.491
* β*-blocker, *n* (%)	22 (34.9%)	29 (36.7%)	23 (36.5%)	0.973
ARB, *n* (%)	25 (4.0%)	39 (4.9%)	24 (3.8%)	0.331
CCB, *n* (%)	39 (61.9%)	58 (73.4%)	39 (61.9%)	0.237
Aldactone, *n* (%)	15 (2.4%)	23 (2.9%)	11 (1.7%)	0.27
Vasodilator, *n* (%)	6 (9.5%)	5 (6.3%)	3 (4.8%)	0.612
Diuretics, *n* (%)	2 (3.2%)	6 (7.6%)	7 (11.1%)	0.215
Pre-operative				
Mean serum potassium, mEq/L (SD)	3.5 (0.6)	3.6 (0.6)	3.9 (0.5)	0.0006
Mean serum creatinine, mg/dL (SD)	0.9 (0.5)	0.9 (0.3)	0.9 (0.3)	0.5267
Mean plasma aldosterone, ng/dl (SD)	57.0 (32.3)	60.5 (49.9)	42.4 (24.4)	0.0159
Mean PRA, ng/ml/hr (SD)	0.5 (0.9)	0.5 (0.7)	0.5 (0.9)	0.7957
Mean ARR (SD)	756.9 (1958.9)	1136.8(2425.1)	971.9 (2100.8)	0.5915
Post-operative 6th month				
Mean serum potassium, mEq/L (SD)	4.4 (0.5)	4.4 (0.4)	4.1 (0.4)	0.0006
Mean serum creatinine, mg/dL (SD)	1.2 (0.7)	1.0 (0.4)	1.0 (0.5)	0.0755
Mean plasma aldosterone, ng/dl (SD)	26.2 (19.5)	31.8 (21.8)	35.0 (23.3)	0.1022
Mean PRA, ng/ml/hr (SD)	3.6 (3.0)	2.1 (2.2)	1.9 (3.0)	0.0023
Mean ARR (SD)	48.3 (117.8)	94.3 (278.3)	196.1 (608.9)	0.118
Post-operative 12th month				
Mean serum potassium, mEq/L (SD)	4.3 (0.5)	4.3 (0.4)	4.2 (0.4)	0.113
Mean serum creatinine, mg/dL (SD)	1.2 (0.9)	1.0 (0.4)	1.0 (1.0)	0.1545
Mean plasma aldosterone, ng/dl (SD)	27.7 (20.1)	39.5 (25.2)	32.4 (16.1)	0.01
Mean PRA, ng/ml/hr (SD)	3.3 (2.3)	3.5 (4.8)	2.2 (3.0)	0.1325
Mean ARR (SD)	88.1 (443.9)	123.6 (425.7)	304.7 (1078.8)	0.2253

*PA* primary aldosteronism, *SD* standard deviation, *BMI* body mass index, *DM* diabetes mellitus, *CAD* coronary artery disease, *MI* myocardial infarction, *CVA* cerebrovascular accident, *HTN* hypertension, *CCI* Charlson comorbidity index, *AVS* adrenal venous sampling, *LI* lateralization index, *ACEI* angiotensin-converting enzyme inhibitors, *ARB* angiotensin receptor blockers, *CCB* calcium channel blockers, *PRA* plasma renin activity, *ARR* aldosterone-renin ratio

The overall complete clinical success rate was 200/390 (51.28%) in all patients with lateralized PA, with the PA patients of the black group having the highest complete clinical success rate (41/63 [65.08%]), when compared to that of the grey (40/79 [50.63%]) or white group (17/63 [26.98%]), as shown in Table 2 (*p* < 0.0001). The overall partial clinical success rate was 38.54% in lateralized PA patients, with 28.57% in the black group, 34.18% in the grey group, and 53.97% in the white group. There was no statistically significant difference in the biochemical outcomes between the three groups, with a complete biochemical success rate of 87.30%, 82.28%, and 80.95%, respectively (*p* = 0.595) (Table 3).

**Table 2:** PASO clinical success of the PA patients after unilateral adrenalectomy in the black, grey, and white groups

	Black (*n* = 63)	Grey (*n* = 79)	White (*n* = 63)	*p*-value
**Complete**	41 (65.08%)	40 (50.63%)	17 (26.98%)	<0.0001^+^
*KCNJ5* mutation(+)	26/41	18/40	0/17	*
**Partial**	18 (28.57%)	27 (34.18%)	34 (53.97%)	0.008^#^
*KCNJ5* mutation(+)	10/18	8/27	3/34	NA
**Absent**	4 (6.35%)	12 (15.19%)	12 (19.05%)	0.089
*KCNJ5* mutation(+)	2/4	4/12	0/12	NA

*PASO* primary aldosteronism surgical outcome, *PA* primary aldosteronism

^+^Post-hoc analysis revealed significant differences between the black and white (*p* < 0.0001) and the grey and white (*p* = 0.012) groups. *p < 0.0001. ^#^Post-hoc analysis revealed significant differences between the black and white (*p* = 0.010) and the grey and white (*p* = 0.046) groups

**Table 3:** PASO biochemical success of the PA patients after unilateral adrenalectomy in the black, grey, and white groups

	Black (*n* = 63)	Grey (*n* = 79)	White (*n* = 63)	*p*-value
**Complete**	55 (87.30%)	65 (82.28%)	51 (80.95%)	0.595
*KCNJ5* mutation(+)	34/55	23/65	3/51	*
**Partial**	3 (4.76%)	5 (6.33%)	4 (6.35%)	1.0
*KCNJ5* mutation(+)	3/3	3/5	0/4	NA
**Absent**	5 (7.94%)	9 (11.39%)	8 (12.70%)	0.669
*KCNJ5* mutation(+)	1/5	4/9	0/8	NA

*PASO* primary aldosteronism surgical outcome, *PA* primary aldosteronism

*p < 0.0001

Among the patients with a complete clinical or complete biochemical success, more PA patients in the black group had *KCNJ5* mutation (*p* < 0.0001). Furthermore, in the patients with absent clinical success after undergoing a total adrenalectomy, *KCNJ5* mutation was noted in 2/4 patients in the black group, 4/12 patients in the grey group, and 0/12 patients in the white group (Tables 2 and 3).

Patients in all groups had a comparable preoperative blood pressure. Follow up mean blood pressures at the post-total adrenalectomy 6th and 12th months significantly decreased when compared to the preoperative blood pressures. In addition, the PA patients in the grey and white groups seem to have less and slower recovery of their hypertension after undergoing a unilateral adrenalectomy (Tables 4 and 5).

**Table 4:** Mean blood pressure in the preoperative, postoperative 6th, and postoperative 12th month of the PA patients in the black, grey, and white groups

	Black (*n* = 63)	Grey (*n* = 79)	White (*n* = 63)	*p*-value
**Pre-operative mean blood pressure, mm Hg (SD)**				
Systolic	157.9 (22.7)	154.9 (19.4)	150.2 (19.0)	0.1059
Diastolic	95.5 (14.2)	92.3 (12.3)	90.2 (13.5)	0.0869
**Post-operative (6th** **month) mean blood pressure, mmHg (SD)**				
Systolic	135.5 (21.3)	137.8 (16.9)	140.0 (17.9)	0.4695
Diastolic	85.0 (12.0)	85.3 (11.6)	87.1 (12.8)	0.6049
**Post-operative (12th** **month) mean blood pressure, mmHg (SD)**				
Systolic	130.6 (19.4)	131.1 (14.9)	140.6 (18.8)	0.0027^+^
Diastolic	81.0 (12.1)	80.8 (9.9)	86.6 (13.7)	0.0111^#^

*PA* primary aldosteronism, *SD* standard deviation

^+^Post-hoc analysis revealed significant differences between the black and white (*p* = 0.009) and the grey and white (*p* = 0.007) groups. ^#^Post-hoc analysis revealed significant differences between the black and white (*p* = 0.043) and the grey and white (*p* = 0.017) groups

**Table 5:** Changes in blood pressure of the PA patients in the black, grey, and white groups at the postoperative 6th and 12th months

**Post-operative (6th month) change in mean blood pressure, mmHg (SD)**						
	Black (*n* = 55)		Grey (*n* = 73)		White (*n* = 51)	
Systolic	22.0 (2.5)	*p* < 0.0001	17.1 (2.2)	*p* < 0.0001	11.2 (2.1)	*p* < 0.0001
Diastolic	11.2 (1.7)	*p* < 0.0001	6.5 (1.5)	*p* < 0.0001	2.8 (1.4)	*p* = 0.0273
**Post-operative (12th** **month) change in mean blood pressure, mmHg (SD)**						

	Black (*n* = 53)		Grey (*n* = 76)		White (*n* = 57)	
Systolic	29.7 (2.9)	*p* < 0.0001	23.4 (2.2)	*p* < 0.0001	10.1 (2.2)	*p* < 0.0001
Diastolic	15.4 (2.1)	*p* < 0.0001	11.6 (1.3)	*p* < 0.0001	3.6 (1.4)	*p* = 0.0049

*PA* primary aldosteronism, *SD* standard deviation

## Discussion

The proportion of classical and non-classical and the percentage of co-existing mAPM/mAPN in clinically diagnosed lateralized PA are reported in this study. In a study of patients diagnosed with a single APA, Jeschke et al. reported successful treatment of hypertension and hyperaldosteronism with low complication rate [5]. However, our results show that, among the patients with lateralized PA, only 63 (30.73%) of the resected adrenal glands contained solitary APA or a dominant APN with positive CYP11B2 staining. Despite previous study reporting successful treatment of hypertension following resection of the involved tumor, our results revealed that, if only partial adrenalectomy were performed among these lateralized PA patients, we could completely remove the aldosterone-producing part among the PA patients in the black group, while leaving the aldosterone-producing parts of those (about 69.3%) in the other two groups in the remaining ipsilateral adrenal gland. Such a selective surgical approach could very likely yield a suboptimal treatment success rate in treating lateralized PA, and thus the majority of lateralized PA patients may not benefit after receiving a partial adrenalectomy.

Kitamoto et al. reported their experience using segmental selective AVS to identify the intra-adrenal aldosterone activity [23]. If the classical type (black group) is confirmed using segmental adrenal tributary sampling, partial adrenalectomy might potentially be acceptable. However, several factors, such as variable accompanying cortisol levels, may affect the data interpretation and lead to possible underdiagnoses of functioning mAPN/mAPM [24].

For inoperable patients, alternative ablative treatments may be considered, including radiofrequency ablation (RFA), cryoablation, and microwave ablation [25–28]. Among these methods, RFA is the most commonly used for adrenal tumors due to its small ablation zone and excellent clinical and biochemical outcomes for functional benign adrenal tumors, such as APA, cortisol-secreting adenomas, and pheochromocytomas [29]. Common complications include hypertensive crisis, bleeding, infection, bowel injury, pneumothorax, and adrenal insufficiency. Both RFA and partial adrenalectomy can be useful in avoiding adrenal insufficiency. However, with the theory of HISTALDO having the possibilities of co-existing mAPM/mAPN that could still secrete excess plasma aldosterone, even if the visible tumoral mass is well treated with an ablation therapy, it still carries the potential risk of incomplete treatment, like the case for partial adrenalectomy. Further prospective, randomized studies are needed to compare the clinical and biochemical success rates and remaining adrenal function outcomes of RFA or partial adrenalectomy versus unilateral total adrenalectomy if such therapies would like to be applied to all lateralized PA patients [26–34].

In our study, biochemical success rates were comparable across the three groups, but complete clinical success rates were lower in the grey and white groups. The comparable biochemical success rates among the HISTALDO groups confirmed the accuracy of our lateralized PA diagnosis. However, patients in the grey and white groups seem to have less and slower recovery of their clinical outcomes, despite having undergo unilateral total adrenalectomy rather than partial adrenalectomy. Factors such as age, comorbidities, and longer duration of hypertension may contribute to poorer clinical outcomes after unilateral adrenalectomy for the lateralized PA patients. Notably, patients in the black group were significantly younger than those in the other two groups (*p* = 0.0125), as shown in Table 1. While hypertension duration appeared longer in the grey and white groups, this difference was not statistically significant. Contralateral suppression during AVS was observed in three patients in the black group, two in the grey group, and none in the white group. All three black group patients achieved complete clinical and biochemical success, whereas one grey group patient achieved complete clinical success and the other achieved partial clinical success. These differences may explain why the complete clinical success rates were lower in the grey and white groups, and why the grey and white groups seem to have less and slower recovery of their clinical outcomes, despite having undergo unilateral total adrenalectomy. However, more research is needed for further clarification.

Furthermore, rarely did the literature discuss PA patients with PASO partial clinical success actually still benefit from unilateral adrenalectomy (better controlled blood pressure, fewer antihypertensive medications; even though not normotensive without medications; according to the definition of partial clinical success), and thus could still be considered as an “optimistic outcome” after unilateral adrenalectomy for those lateralized PA patients. Despite the complete clinical success being better achieved in the black group, our results showed that lateralized PA patients in all groups have a combo clinical and biochemical optimistic outcomes rates (rates of complete success plus partial success) above 80% after undergoing unilateral adrenalectomy; justifying the benefits of unilateral total adrenalectomy for lateralized PA patients. But if only partial adrenalectomy (tumorectomy) were performed for lateralized PA patients of the grey and white groups, the mAPM/mAPN or APDH that secrete excess aldosterone would not be removed, then the optimistic outcomes in these PA patients would not be observed as currently seen. Thus, we conclude that in clinically lateralized PA patients, no matter which group they are in the HISTALDO classification, they would benefit from unilateral total adrenalectomy (no matter either clinical or biochemical outcomes are concerned).

It is known that mutations in the *KCNJ5* gene are associated with APA, and are the most frequently known alterations in patients with PA [35]. However, our results showed that having a positive *KCNJ5* mutation does not absolutely assure surgical success, even in the black (3.17% clinical failure and 1.59% biochemical failure) or grey group (5.06% clinical failure and 5.06% biochemical failure) of the lateralized PA patients.

This study has limitations. First, there were 185 lateralized PA specimens technically too challenging to be classified to a specific HISTALDO group, primarily due to inadequate tissue sampling. This issue arose because, in the past, only our specialized research lab conducted CYP11B2 immunohistochemical staining. Eventually, the pathology department in our hospital adopted it as a routine procedure for all clinically diagnosed PA adrenal specimens. Furthermore, in the early years of our TAIPAI cohort, either only the tumoral parts were collected for research, or the specimens included just the tumor and a small amount of adjacent normal-appearing adrenal tissue. As a result, we could not classify these 185 patients in the final analysis. However, if the distribution of HISTALDO classifications among these patients is consistent, we hypothesize that the proportions across groups should be similar. Second, only the *KCNJ5* gene was analyzed in our study. We found that having a positive *KCNJ5* mutation does not necessarily guarantee surgical success. This observational cohort requires further evaluation to clarify its clinical and pathophysiological significance.

### Perspective of Asia

While the presence of a *KCNJ5* somatic mutation has been identified as an independent predictor of hypertension remission following unilateral adrenalectomy in patients with lateralized PA, its prevalence differs between Asian and European populations [35–38]. Furthermore, our findings suggest that having a positive *KCNJ5* mutation does not guarantee surgical success. Therefore, further studies are needed to confirm the prognostic value of *KCNJ5* mutations in predicting hypertension remission after adrenalectomy for lateralized PA in other ethnic groups.

## Conclusion

Our results show that partial adrenalectomy for lateralized PA patients may potentially lead to a significantly lower treatment success rate, no matter the criteria of success is considered as “complete success only” or “both complete and partial success”. Thus, based on reasonable inferences from the pathological analysis results using the well-defined HISTALDO classification—which clearly identifies the functionally active aldosterone-producing components of the diseased adrenal gland—we speculate that unilateral partial adrenalectomy is not a good surgical option for the majority of lateralized PA patients. Furthermore, patients in all groups demonstrate a clinical and biochemical “optimistic outcomes” rate above 80% (considering complete success plus partial success as optimistic outcomes). Therefore, we conclude that in clinically lateralized PA patients, no matter which group they are in the HISTALDO classification, they would benefit from unilateral total adrenalectomy. In addition, having a positive *KCNJ5* mutation does not assure surgical success. All these observations require further, fully prospective evaluation to elucidate its clinical and pathophysiological significance.
